# Attentional Demands Influence Vocal Compensations to Pitch Errors Heard in Auditory Feedback

**DOI:** 10.1371/journal.pone.0109968

**Published:** 2014-10-10

**Authors:** Anupreet K. Tumber, Nichole E. Scheerer, Jeffery A. Jones

**Affiliations:** Psychology Department and Laurier Centre for Cognitive Neuroscience, Wilfrid Laurier University, Waterloo, Ontario, Canada; University of Texas Health Science Center at San Antonio, Research Imaging Institute, United States of America

## Abstract

Auditory feedback is required to maintain fluent speech. At present, it is unclear how attention modulates auditory feedback processing during ongoing speech. In this event-related potential (ERP) study, participants vocalized/a/, while they heard their vocal pitch suddenly shifted downward a ½ semitone in both single and dual-task conditions. During the single-task condition participants passively viewed a visual stream for cues to start and stop vocalizing. In the dual-task condition, participants vocalized while they identified target stimuli in a visual stream of letters. The presentation rate of the visual stimuli was manipulated in the dual-task condition in order to produce a low, intermediate, and high attentional load. Visual target identification accuracy was lowest in the high attentional load condition, indicating that attentional load was successfully manipulated. Results further showed that participants who were exposed to the single-task condition, prior to the dual-task condition, produced larger vocal compensations during the single-task condition. Thus, when participants’ attention was divided, less attention was available for the monitoring of their auditory feedback, resulting in smaller compensatory vocal responses. However, P1-N1-P2 ERP responses were not affected by divided attention, suggesting that the effect of attentional load was not on the auditory processing of pitch altered feedback, but instead it interfered with the integration of auditory and motor information, or motor control itself.

## Introduction

Proficient motor control is achieved by using sensory feedback to plan, execute, and regulate motor movements [1]. This is particularly true for speech motor control, which relies on auditory feedback for the regulation of ongoing and future speech motor commands [2], [3]. In everyday life, speakers receive auditory feedback while simultaneously processing information from other modalities. Since attention is a limited resource, it must be divided amongst the input from different sensory modalities based on the processing demands and encoding requirements imposed by these sensory modalities [4]. In order to understand how auditory feedback facilitates fluent speech motor control, particularly when speech errors are encountered, it is important to understand how attention modulates the processing of auditory feedback during ongoing speech.

The multiple resource theory of divided attention states that when performing two tasks simultaneously, the degree to which performance will decline on each task, compared to when each task is completed in isolation depends on: the resource demands of each of the two tasks, the similarity between the two tasks, and the allocation of resources between the two tasks [5]. Studies examining cross-modal (e.g., visual and auditory) attention have argued for separate, but linked attentional systems [6], [7]. When simple stimuli are being processed, separate attentional resources are utilized by each modality, eliminating any interference that may occur as a result of simultaneously processing stimuli in different modalities [8], [9], [10]. However, when cross-modal stimuli are complex, and the attentional load is increased, attending to one stimulus modality may interfere with the processing of a second stimulus in a different modality. For example, when participants performed a visual discrimination task where they were required to adjust the length of the arms of a cross-shape, they were less likely to notice a binaurally presented tone [11]. Together, these theories suggest that increasing one’s attentional load during ongoing speech, by introducing a secondary task, may interfere with the processing of auditory feedback.

The importance of auditory feedback for maintaining fluent speech has been demonstrated by individuals who have been deafened post-lingually, and experienced a gradual deterioration in the quality of their speech [12]. However, since there are inherent delays involved in processing auditory feedback, a feedforward system driven by internal models must also play a role in fluent speech production, as strict reliance on auditory feedback would result in delayed and inarticulate speech [2], [3]. That being said, in order to ascertain the role of auditory feedback during ongoing speech, the frequency-altered feedback (FAF) paradigm is often utilized [13], [14], [15]. As part of this paradigm, participants produce vocalizations while their fundamental frequency (F0), or vocal pitch, is shifted upwards or downwards and instantaneously presented back to them through headphones. When the F0 of an individual’s auditory feedback is altered, they tend to compensate, or shift their voice in the opposite direction of the manipulation. Since the compensatory response is often only a fraction of the size of the manipulation [13], [14], [16], [17], [18], [19], it has been suggested that it is an automatic response intended to correct for small production errors [20], [21]. However, it is currently unclear whether attention load modulates this reflexive-like response.

In addition to investigating vocal responses, auditory cortical responses to FAF recorded using electroencephalography (EEG) can provide information regarding the underlying neural mechanisms of speech motor control. The P1, N1, and P2 event-related potentials (ERPs) are reliably elicited by FAF [18], [19], [22]. The P1 is proposed to reflect the early detection of changes in auditory feedback, as previous research has demonstrated that it is elicited in an all-or-nothing manner when FAF perturbations are under 400 cents (100 cents is equivalent to a semitone) [18], [22]. On the other hand, the N1 is thought to reflect pre-attentive error-detection, where auditory feedback is compared to a sensory prediction produced by the motor system during the execution of speech motor commands [23]. In line with this notion, Scheerer et al. [18] found that smaller feedback perturbations (less than 250 cents) evoked similarly sized N1 responses, while larger (400 cent) perturbations resulted in significantly larger N1 responses. Based on these findings, it was suggested that the N1 ERP component specifically reflects whether a feedback error is physiologically feasible, and thus likely to be internally generated, or excessively deviant, and thus likely to be externally generated. All feedback alterations perceived as physiologically feasible, elicit small N1 responses, compared to feedback alterations perceived as physiologically implausible, which generate larger N1 responses [18]. The third ERP component commonly elicited by FAF, the P2, has been shown to increase linearly as the size of the feedback perturbations increases [18], leading to the suggestion that the amplitude of the P2 component reflects the size of the speech production error [22], [24]. Although researchers are just beginning to understand how FAF modulates the P1, N1, and P2 ERP components, their sensitivity to FAF makes them ideal for assessing the influence of attention on the processing of auditory feedback during ongoing speech.

Although it is unclear how FAF modulates these ERPs under divided attention, when elicited by other forms of auditory stimuli, auditory ERPs have shown sensitivity to divided attention. In particular, the N1 component is often enhanced when participants attend to an auditory stimulus, relative to passive listening of the same stimulus [25], [26], [27], [28], [29]. Specifically, Choi and colleagues [25] found that when comparing attended and unattended auditory streams, attentional gains to attended auditory stimuli were associated with an approximate 10 dB increase in loudness, compared to auditory stimuli in the unattended auditory stream. These findings suggest that focused auditory attention results in larger N1 responses to auditory stimuli, and increases the perceived loudness of auditory stimuli. On the other hand, P1 and P2 amplitudes are rarely modulated by selective attention toward an auditory channel (e.g., Choi et al., [24], Coch et al., [26]). Increases in the latency of slow negative ERPs related to the N1 have also been found when attention is divided between two auditory channels, compared to when attention is oriented to a specific channel, which has been attributed to increased processing demands under divided attention [30]. Together these results suggest that the P1-N1-P2 ERP responses may be modulated by divided attention.

For the current experiment, we used a dual-task paradigm to investigate whether divided attention impacts the compensatory vocal responses and ERPs elicited by FAF. In order to reduce the allocation of attentional resources to auditory feedback during the FAF task, participants simultaneously monitored a rapid serial visual presentation (RSVP) of letters. The RSVP contained target letters, which participants identified and later reported. Attentional load was manipulated by varying the rate of the RSVP. Increasing the rate of the RSVP decreased the inter-stimulus interval (ISI) between letters, which modulated the perceptual load by increasing the number of stimuli. As a result of the increased number of stimuli, participants had to process more irrelevant information, which directly impacted the perceptual selection of relevant information, and thus increased the overall attentional demand of the task (see Chun & Wolfe, [31] and Lavie, Hirst, de Fockert, & Viding, [32], for a review).

Since decreasing the ISI of a RSVP of letters has been shown to increase attentional load, we expected that increasing the rate of the RSVP, would reduce participants’ abilities to identify the target letters. Furthermore, we expected that as the rate of the RSVP increased, more attention would be allocated to the visual task, which would reduce the saliency of the FAF, and result in smaller and slower compensatory responses. With regard to the ERP responses, since the P1 is thought to reflect the basic detection of FAF [18], we expected that as attentional load increased, and the FAF became less salient, P1 amplitudes would decrease. Similarly, since previous research has shown that attending to an auditory stimulus results in larger N1s, we expected that N1s would be larger in the single-task condition as more attention would be allocated to the processing of auditory feedback, relative to the dual-task, where attention would be divided between the auditory feedback and the visual stream. On the other hand, since the size of the FAF perturbations were not manipulated in this experiment, and the P2 component is thought to play a role in assessing the size of FAF perturbations, we did not predict changes in P2 amplitudes as a function of attention load. However, we did predict that P1-N1-P2 latencies would be later under divided attention, reflecting slower processing under increased attentional load.

## Methods

### Participants

Sixty-five participants between the ages of 16 and 38 years (*M* = 21.51 years, *SD* = 4.81; 42 females and 23 males) participated. Vocal and behavioural responses were recorded from all 65 participants, while ERP responses were also recorded from a subset of 33 right-handed participants (M = 19 years, SD = 1.37; 21 females and 12 males). All participants were Canadian-English speakers who did not speak a tonal language, with the exception of one participant who spoke a tonal language, but identified English as their primary language. This tonal language speaker did not show any differences in vocal compensations nor ERP responses to the FAF perturbations compared to the other non-tonal language-speaking participants. All participants also had normal or corrected to normal vision, had not been diagnosed with attention deficit (hyperactivity) disorder (ADD, ADHD), epilepsy (or had a family history of seizures), visual deficits that could not be amended by corrective lenses, and did not have a speech or language disorder.

### Ethics Statement

All participants provided written informed consent and received financial compensation or course credit for their participation in this study. All procedures were approved by the Wilfrid Laurier University Research Ethics Board and were in accordance with the World Medical Association 2013 Declaration of Helsinki.

### Procedure

Participants produced 198 vocalizations of the vowel sound/a/, across two conditions, while exposed to a RSVP. Each vocalization was randomly perturbed downward 50 cents for 200 ms. During the single-task condition, participants produced nine practice vocalizations, followed by a block of 45 vocalizations where the participants’ only goal was to produce a steady/a/sound. During the dual-task condition, participants produced nine practice vocalizations, followed by 3 blocks of 45 vocalizations, where participants were also required to attend to a RSVP and answer questions about the visual stream.

For both experimental conditions, each visual stream had two targets: 1. a randomly selected white letter from the alphabet (excluding letter “X”); and 2. an “X,” that occurred pseudorandomly before or after the white letter. All letters in the stream were capitalized and in black font with the exception of the white target letter. Each letter was displayed at the same location in the center of a grey field, where the varied ISI was seen as a uniform grey field. For the single-task trials, each letter stream ended with a blank grey field.

**Figure 1:**
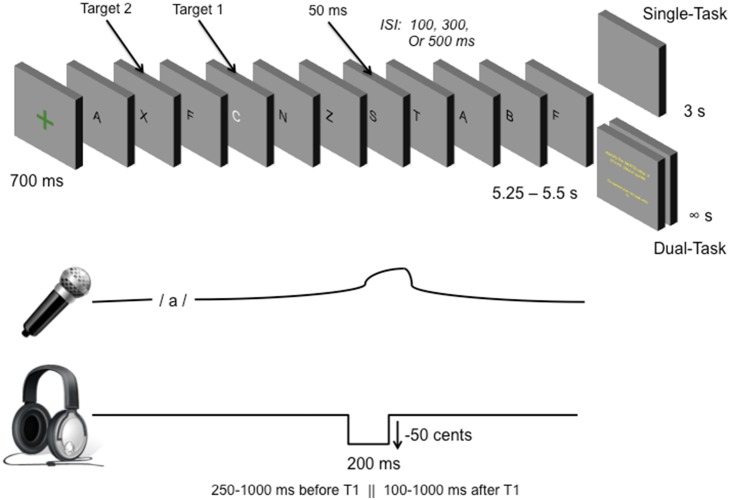
Experimental paradigm. Participants observed a rapid serial visual presentation (RSVP) of letters with two targets (a white letter, and an “X”). In the single-task condition, participants observed a blank screen for 3 s before the next trial. For the dual task condition, participants answered two questions at the end of each trial; they were asked to identify the white letter and whether the “X” appeared before or after the white letter. Attentional load was manipulated by varying the inter-stimulus-interval across trials: 500 ms for the low attentional load, 300 ms for the intermediate attentional load, and 100 ms for the high attentional load. All trials were approximately 5.5 s long. Participants vocalized the/a/sound during the letter stream in both conditions, while listening to their auditory feedback, which was perturbed downward 50 cents for 200 ms either 250–1000 ms before target 1, or 100–1000 ms after target 1.

The presentation rate of the RSVP was manipulated to impose a high, intermediate, and low attentional load. The RSVP started with a green fixation cross that lasted for 750 ms, followed by a stream of successively presented letters. Each trial was approximately 5.5 s in duration. Each letter appeared for 50 ms, and each trial occurred with an ISI of either 100, 300, or 500 ms (high, intermediate, or low attentional load, respectively). High attentional load trials were 5.4 s in duration and consisted of 37 letters, with an ISI of 100 ms. The white letter appeared at random between the 4^th^ and the 37^th^ letter, and the “X” randomly occurred between the 1^st^ and 15^th^ letter before or after the white letter (minimum 2^nd^ place, maximum 36^th^ place in the visual stream). Intermediate attentional load trials were 5.4 s in duration and consisted of 16 letters in the visual stream, with an ISI of 300 ms. The white letter appeared at random between the 4^th^ and 16^th^ place in the letter stream, while the “X” occurred at random between the 1^st^ and 12^th^ letter before or after the white letter (minimum 2^nd^ letter and maximum 16^th^ letter). Low attentional load trials were 5.5 s in duration, with 11 letters in the stream and an ISI of 500 ms. The white letter occurred randomly between the 4^th^ and 11^th^ place in the visual stream, and the “X” occurred at random between the 1^st^ and 15^th^ letters from the white letter (minimum 2^nd^ letter in the stream, maximum 11^th^). Figure 1 depicts the paradigm. Each presentation rate occurred an equal number of times in both the single- and dual-task trials, but was pseudo-randomly presented throughout the experiment. The arrangement of the dual-task condition and the single-task condition was counterbalanced across participants.

During the single-task condition, participants were instructed to attend to the RSVP, as the start of the RSVP was their cue to start vocalizing, and the termination of the RSVP was their cue to stop vocalizing. During the dual-task condition, participants were instructed to attend to the RSVP and monitor the letter stream for their cue to start and stop vocalizing, but also so they could identify two targets: a white letter, and an “X.” At the end of the letter stream, participants were required to answer two questions about the target letters. The first question appeared on the screen at the end of the trial and stated, “Identify the WHITE letter. If you are unsure, please guess.” The second question, which appeared immediately after the participant’s response to the first question stated, “indicate when the “X” appeared with reference to the white letter.” The participant was required to press a key labelled “YES” if they believed that the “X” appeared before the white letter, and a key labelled “NO” if the believed that the “X” appeared after the white letter. During each vocalization, the FAF perturbation occurred either 250–1000 ms before the “X,” or 100–1000 ms after the “X.” Emphasis was placed on both maintaining a steady vocalization and on responding accurately to the two questions at the end of the trial. Since participants could take as much time as they needed to respond to the two questions, participants moved on to subsequent trials at their own pace during the dual-task trials, whereas single-task trials occurred with an inter-trial interval of 3000 ms. The total duration of the experiment ranged from 40 to 60 minutes, and depended on the participant’s reaction time to the questions during the dual-task blocks, and the duration of breaks between blocks.

### Apparatus

Participants were seated 76 cm from a 15-inch LCD monitor in an electrically shielded booth (Raymond EMC, Ottawa, ON, Canada) and were fitted with a HydroCel GSN 64 1.0 Cap (Electrical Geodesics Inc., Eugene, OR, USA), Etymotic ER-3 insert earphones (Etymotic Research, Elk Grove Village, IL, USA), and an earset microphone (Countryman Isomax, IL, USA). The presentation of visual stimuli and shift onsets and offsets were controlled by Max/MSP 6 (Cycling’ 74, San Francisco, CA). Keyed behavioral responses to questions were also recorded using Max/MSP 6, using a standard keyboard with labeled keys. During the experiment, voice signals were sent to a mixer (Mackie Onyx 1200, Loud Technologies, Woodinville, WA, USA), then to a digital signal processor (DSP; VoiceOne, T.C. Helicon, Victoria, BC, Canada), which altered the F0 of the voice signal. This process introduced a ∼10 ms delay in the feedback signal, that was then presented back to the participant through headphones as auditory feedback. The unaltered voice signal was digitally recorded (TASCAM HD-P2, Montebello, CA, USA) at a sampling rate of 44.1 kHz for later analysis.

### Analysis

#### Behavioural Analysis

For each trial, records were kept of the trial’s attentional load condition, the white letter in the stream, whether the “X” appeared before or after the white letter, the participants’ keyed responses and reaction time to question one, as well as the participants’ keyed responses and reaction time to question two. Participants who did not answer either of the questions according to the instructions were excluded from the analysis. One participant’s answers were excluded from the white letter identification analyses for this reason. Accuracy and reaction time for questions one and two were averaged for each of the three categories of attentional load in the dual-task condition (i.e., low, intermediate, and high attentional load trials). Accuracy for high, intermediate, and low attentional load trials were then averaged across all participants for each question. Only accuracy data were examined since response accuracy was emphasized during the participant instructions.

#### Vocal Analysis

Each participant’s unaltered voice recording was segmented into separate vocalizations. The swipe algorithm [33] was used to determine F0 values for each vocalization. The vocalizations were then segmented based on the onset of the perturbation, where F0 values were normalized to a baseline period 200 ms prior to the onset of the perturbation. This normalization was achieved by converting Hertz values to cents using the formula: cents = 1200 (LOG2(F/B)), where F is the F0 value in Hertz, and B is the mean frequency of the baseline period. Cents values were calculated for the baseline period (200 ms prior to the start of the perturbation), and 1000 ms after the perturbation.

Missing and incomplete vocalizations were excluded from the statistical analysis. Participants with more than 67% (i.e., 30 out of 45) rejected trials for any of the four attentional load conditions (i.e., dual task-high, dual task-intermediate, dual task-low, and single task attentional load trials) were removed from further analysis. A total of 11 participants were excluded from the experiment due to F0 tracking issues. For the 54 remaining participants, an averaged F0 trace for retained trials was constructed for each of the four attentional load conditions. The average number of trials in each condition was 42 for the dual task-high, 40 for the dual task-intermediate, 41 for the dual task-low, and 39 for the single task attentional load trials. Traces were averaged across all participants, for each condition. For each participant, the magnitude of the compensatory response and the latency of the response were assessed. The amplitude of the compensatory response was determined by finding the maximum point at which the participants’ average F0 trace deviated from the baseline mean. Latency was determined as the time at which the maximum peak in the compensatory response occurred.

#### EEG Analysis

EEG signals were recorded from 64-electrodes on the scalp, and referenced online to the vertex (Cz). Signals were bandpass filtered (1–30 Hz) and digitized (12-bit precision) at 1000 samples per second. Impedances were maintained below 50 kΩ for the experiment [34]. EEG-voltage values were re-referenced to the average voltage across all electrodes and then epoched into segments from 100 ms before to 500 ms after the onset of the auditory feedback perturbation. Segments were then analyzed for artifacts and rejected if changes in voltage values exceeded 55 uV over a moving average of 80 ms. A visual inspection of the data was also completed to ensure that segments containing artifacts were excluded from further analysis. Participants with more than 67% (i.e., 30 out of 45) of their trials rejected for any of the four conditions were removed from further analysis. For this reason, three participants were excluded from further analyses, leaving 30 participants for the EEG analysis. For the remaining participants, the average number of trials in each condition was 42 for the dual task-high, 42 for the dual task-intermediate, 41 for the dual task-low, and 41 for the single task attentional load trials. Averaged waveforms were created for each of the four conditions for these participants. All participants’ epochs were then grand-averaged for each condition and baseline corrected.

Six electrodes were included in the analysis: Fz, Cz, F3, C3, C4, and F4. These six electrodes were selected for analysis through visual inspection, having demonstrated the most robust P1-N1-P2 components, and based on previous research, which suggests that front-medial and centro-frontal regions display the most robust responses to pitch shifts [16], [18], [22], [35]. These electrodes were grouped into left (average of F3 and C3), medial (average of Fz and Cz), and right (average of F4 and C4) regions for further analysis. The peak amplitude of the P1 component was extracted from a window between 50 and 100 ms, while the peak amplitude of the N1 component was extracted from a window between 100 and 200 ms, and the peak amplitude of the P2 component was extracted from a time window between 200 and 300 ms. These time windows were determined by visual inspection, based on the latency of the most prominent ERP peaks.

### Statistical Analysis

#### Statistical Analysis of the Behavioural Data

Two separate repeated-measures analysis of variances (RM-ANOVAs) were conducted to look at the influence of attentional load (dual-task high, dual-task intermediate, dual-task low) with block-order (single-task first, dual-task first) as a between-subjects factor, on response accuracy for the identification of the white letter and the placement of the “X”. The Greenhouse-Geisser [36] correction was used in cases where violations of Mauchley’s Assumptions of Sphericity were present. In these cases, the original degrees of freedom were reported for ease of interpretation. Separate Pearson product moment correlations were also conducted for dual-task high, dual-task intermediate, and dual-task low attentional load conditions at an alpha of 0.05 (two-tailed). For each attentional load condition, vocal response magnitude and latency were each correlated with the average accuracy for white letter identification and the average accuracy for “X”-placement.

#### Statistical Analysis of the Vocal Data

Two separate RM-ANOVAs were conducted to assess the influence of attentional load (dual–task high, dual-task intermediate, dual-task low, and single-task) and block-order (single-task first, dual-task first) on vocal response magnitudes and response latencies. Follow-up RM-ANOVAs were run to investigate significant interactions. The Greenhouse-Geisser [36] correction was used in cases where violations of Mauchley’s Assumption of Sphericity were present. In these cases, the original degrees of freedom were reported for the ease of interpretation.

#### Statistical Analysis of EEG Data

Separate two-way RM-ANOVAs were conducted to look at the impact of attentional load (dual-task high, dual-task intermediate, dual-task low, and single-task) and electrode site (left, medial, and right) with the between subject factor of block-order (single-first, dual-first) on P1-, N1-, and P2- amplitudes, and P1-, N1-, P2- latencies. The Greenhouse-Geisser [36] correction was used in cases where violations of Mauchley’s Assumption of Sphericity were identified. In these cases, the original degrees of freedom were reported for ease of interpretation.

## Results

### Behavioural Results

**Figure 2:**
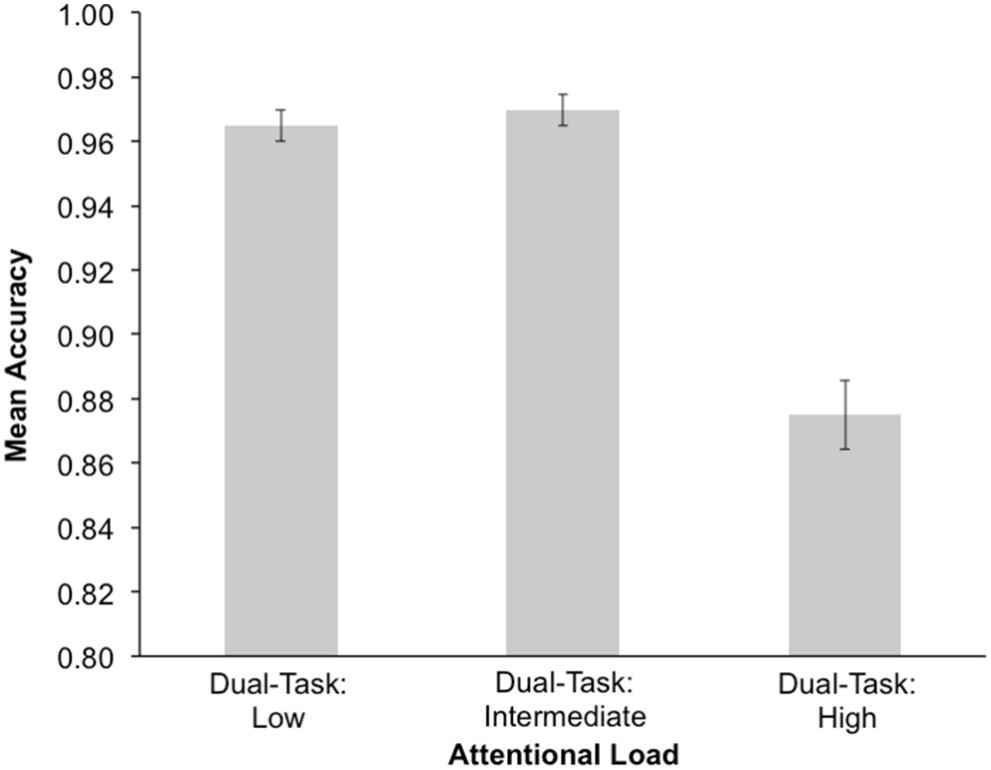
Mean response accuracy for white letter identification during low, intermediate, and high attentional load. Error bars represent standard error.

A RM-ANOVA was conducted to look at the effect of attentional load on white-letter identification accuracy with block-order as a between subjects factor. There was a main effect of attentional load, *F*(2,102) = 78.662, *p*<0.001, *η* ^2^ = 0.607, where white-letter identification was significantly more accurate for the dual-task low attentional load condition, relative to the dual-task high attentional load condition, *p*<0.001. While the dual-task intermediate attentional load condition also elicited higher response accuracy for the white-letter identification, relative to the dual-task high attentional load condition, *p*<0.001 (see Figure 2). The main effect of block-order, *F*(1,51) = 0.686, *p* = 0.411, *η* ^2^ = 0.013, and the interaction between attentional load and block-order were not significant, *F*(2,102) = 0.107, *p* = 0.819, *η* ^2^ = 0.002.

**Figure 3:**
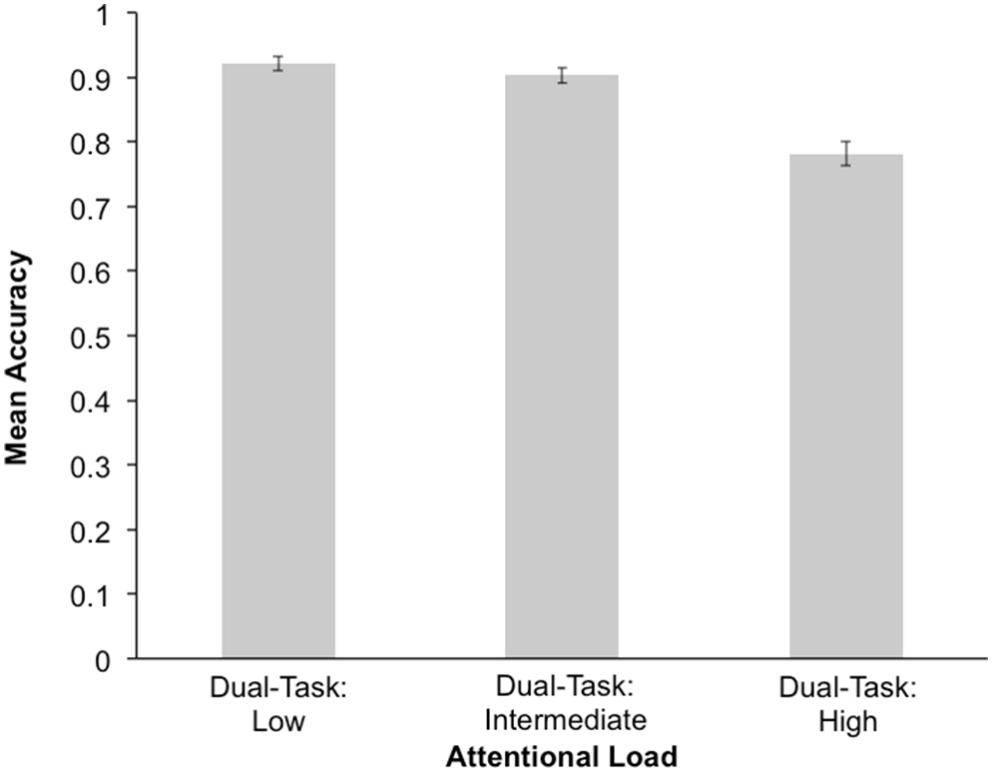
Mean “X”-placement accuracy during low, intermediate, and high attentional load. “X”-placement refers to whether the second target (“X”) in the RSVP was presented before or after the white letter. Error bars represent standard error.

A RM-ANOVA was conducted to look at the effect of attentional load on “X”-placement accuracy, with block-order as a between subjects factor. There was a main effect of attentional load, *F*(2,104) = 106.443, *p*<0.001, *η* ^2^ = 0.672, where accuracy was much higher for the dual-task low attentional load condition, compared to the dual-task intermediate, and dual-task high attentional load conditions, *p*<0.01. In addition, “X”-placement accuracy was also greater for the dual-task intermediate attentional load condition, compared to the dual-task high attentional load condition, *p*<0.001 (see Figure 3). The main effect of block-order, *F*(1,52) = 0.159, *p* = 0.691, *η* ^2^ = 0.003, and the interaction between attentional load and block-order were not significant, *F*(2,104) = 2.454, *p* = 0.107, *η* ^2^ = 0.045.

Pearson-product moment correlations were conducted to look for potential relationships between the magnitude and latency of vocal responses, and the accuracy with which the white-letter and “X”-placement were identified, for the dual-task high, dual-task intermediate, dual-task low attentional load conditions. The correlations between white letter identification accuracy and vocal response magnitude, and white letter identification accuracy and vocal response latency, were not significant, both p>.05. Similarly, the correlations between accuracy for “X”-placement and vocal response magnitude, and accuracy for “X”-placement and vocal response latency, were also not significant, both p>.05.

### Vocal Results

**Figure 4:**
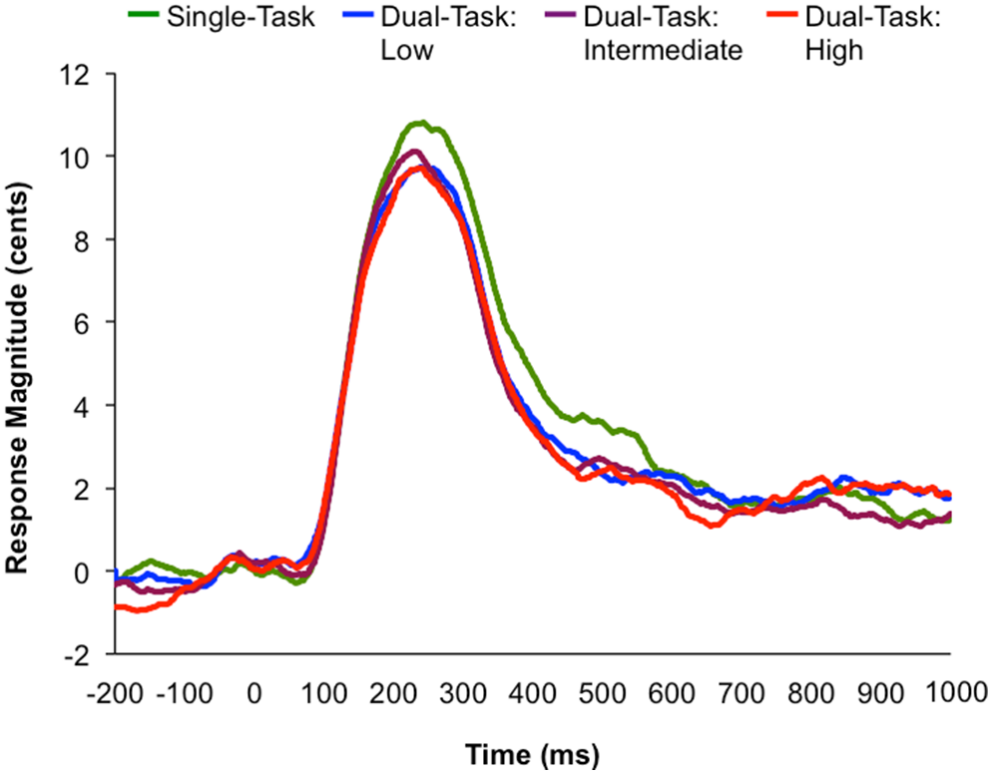
Averaged F_0_ traces for each attentional load condition. Time zero represents the onset of the feedback perturbation.

**Figure 5:**
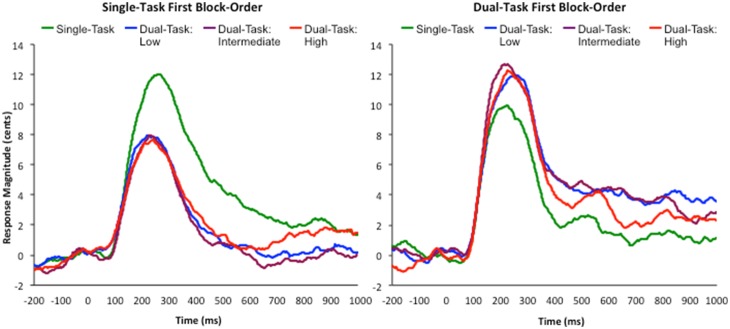
Block-order specific F_0_ traces for each attentional load condition. Left: single-task first block-order; averaged F_0_ traces for the single-task, and dual-task low, intermediate, and high attentional load conditions. Right: dual-task first block-order; averaged F_0_ traces for the single-task, and dual-task low, intermediate, and high attentional load conditions. Time zero on both graphs represents the onset of the feedback perturbation.

A RM-ANOVA was conducted looking at the effect of attentional load on vocal response magnitudes, with block-order as a between subjects factor. The main effect of attentional load on vocal response magnitudes was not significant, *F*(3,156) = 1.175, *p* = 0.321, *η* ^2^ = 0.022 (see Figure 4), nor was the main effect of block-order, *F*(1,52) = 0.573, *p* = 0.453, *η* ^2^ = 0.011. However, a significant interaction was found between attentional load and block-order, *F*(3,156) = 5.782, *p* = 0.003, *η* ^2^ = 0.1 (see Figure 5). Follow-up RM-ANOVAs were conducted to investigate the effect of attentional load on vocal response magnitudes for each block order. A significant main effect of attentional load was found for the single-task first block order, *F*(3,84) = 7.303, *p* = 0.001, *η* ^2^ = 0.207, where the vocal response magnitudes were significantly larger in the single-task condition, compared to all attentional load conditions, *p*<0.02. However, the effect of attentional load on vocal response magnitudes in the dual-task first condition was not significant, *F*(3,72) = 1.083, *p* = 0.362, *η* ^2^ = 0.043.

A RM-ANOVA was conducted to look at the effect of attentional load on vocal response latencies, with block-order as a between subjects factor. The main effect of attentional load was not significant, F(3,156) = 1.684, *p* = 0.173, *η* ^2^ = 0.031, nor was the main effect of block-order, *F*(1,52) = 0.542, *p* = 0.465, *η* ^2^ = 0.01, or the attentional load by block-order interaction F(3,156) = 0.739, *p* = 0.53, *η* ^2^ = 0.014.

### EEG Results

**Figure 6:**
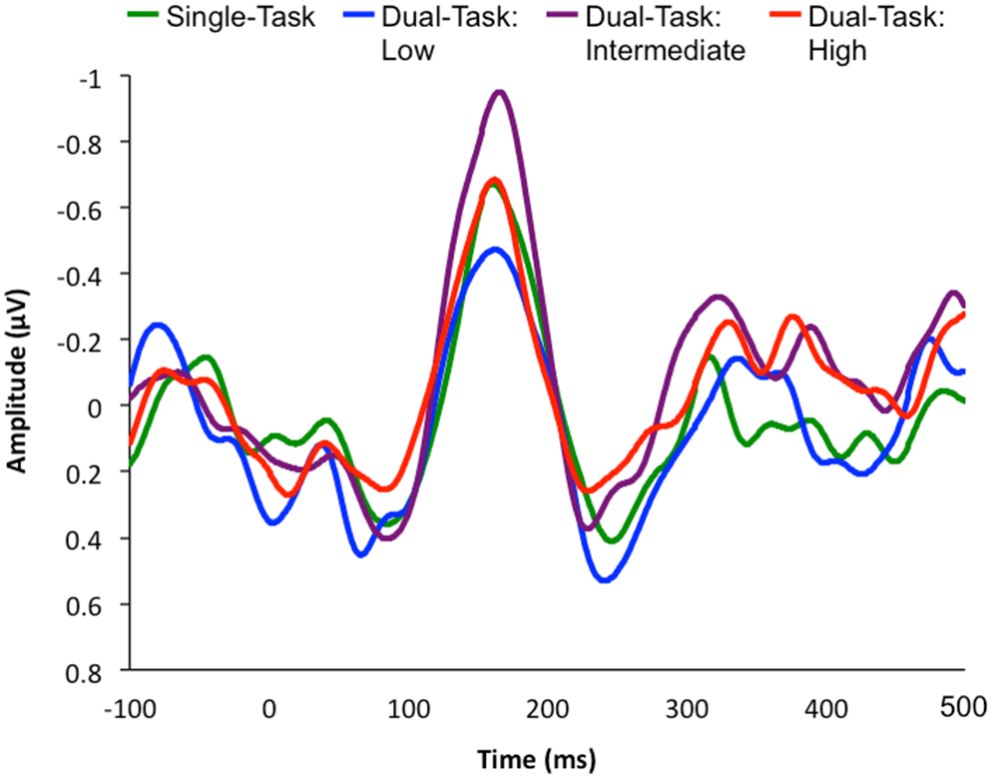
ERP waveforms averaged across all electrode sites for each attentional load condition. The six averaged electrode sites are: F3, C3, Fz, Cz, F4 and C4. Time zero represents the onset of the feedback perturbation.

See Figure 6 for the averaged ERP waveforms across all electrode sites, at dual-task high, dual-task intermediate, dual-task low, and single-task attentional load conditions.

#### P100

A RM-ANOVA was conducted to look at the effect of attentional load, electrode site, and block-order on P100 amplitudes. There was no main effect of attentional load, *F*(3,75) = 1.438, *p* = 0.238, *η* ^2^ = 0.054, no main effect of electrode site, *F*(2,50) = 1.375, *p* = 0.261, *η* ^2^ = 0.052, and no main effect of block-order, *F*(1,25) = 0.009, *p* = 0.926, *η* ^2^<0.001. The attentional load by block-order interaction was also not significant, *F*(3,75) = 0.979, *p* = 0.407, *η* ^2^ = 0.038, as was the electrode site by block-order interaction, *F*(2,50) = 2.067, *p* = 0.15, *η* ^2^ = 0.076, the attentional load by electrode site interaction, *F*(6,150) = 0.281, *p* = 0.945, *η* ^2^ = 0.011, and the three-way interaction between attentional load, electrode site, and block-order, *F*(6,150) = 0.853, *p* = 0.531, *η* ^2^ = 0.033.

A RM-ANOVA was conducted to look at the effect of attentional load, electrode site, and block-order on P100 latencies. There was no main effect of attentional load, *F*(3,75) = 1.349, *p* = 0.265, *η* ^2^ = 0.051, no main effect of electrode site, *F*(2,50) = 0.299, *p* = 0.695, *η* ^2^ = 0.012, and no main effect of block-order, *F*(1,25) = 0.885, *p* = 0.356, *η* ^2^ = 0.034. The attentional load by block-order interaction was not significant, *F*(3,75) = 0.034, *p* = 0.992, *η* ^2^ = 0.001, as was the electrode site by block-order interaction, *F*(2,50) = 0.753, *p* = 0.45, *η* ^2^ = 0.029, the attentional load by electrode site interaction, *F*(6,150) = 0.338, *p* = 0.916, *η* ^2^ = 0.013, and the three-way interaction between attentional load, electrode site, and block-order, *F*(6,150) = 1.246, *p* = 0.286, *η* ^2^ = 0.047.

#### N100

**Figure 7:**
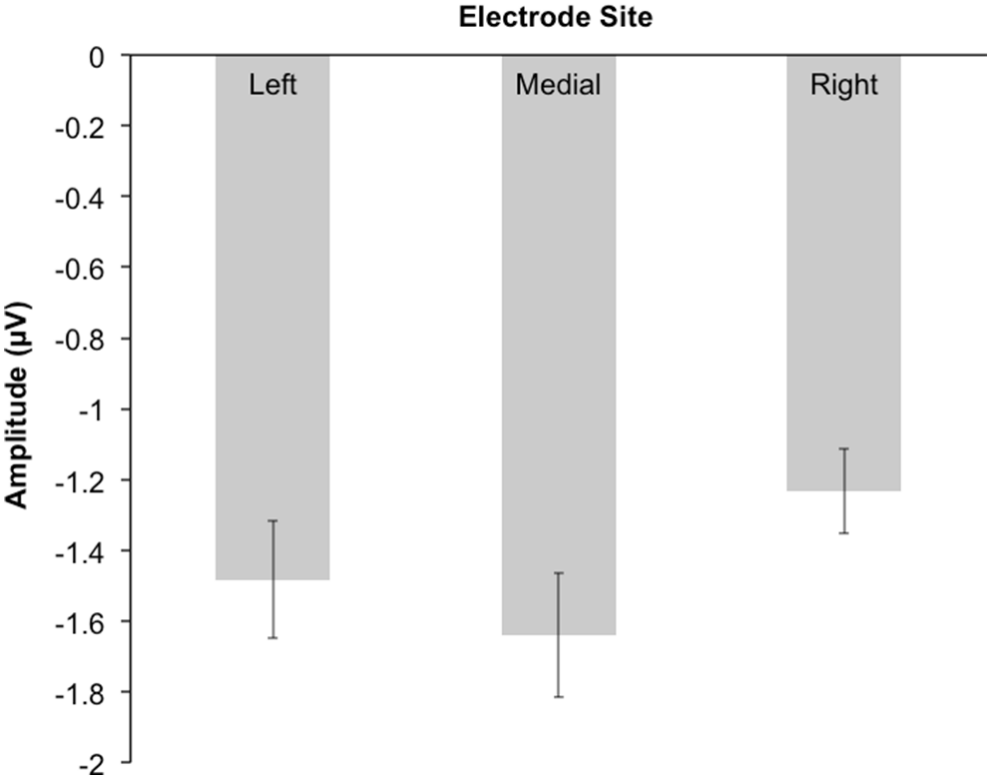
Mean N1 amplitudes for the each electrode site. The left electrode site is the average of the F3 and C3 electrodes; the medial electrode site is the average of the Fz and Cz electrodes; and right electrode site is the average of the F4 and C4 electrodes. Error bars represent standard error.

A RM-ANOVA was performed to look at the effect of attentional load, electrode site, and block-order on N100 amplitudes. There was no significant main effect of attentional load, *F*(3,75) = 2.05, *p* = 0.114, *η* ^2^ = 0.076, nor a significant main effect of block-order, *F*(1,25) = 1.065, *p* = 0.312, *η* ^2^ = 0.041. However, the main effect of electrode site was significant, *F*(2,50) = 4.057, *p* = 0.023, *η* ^2^ = 0.14, such that the right electrode site recorded smaller N1 amplitudes (absolute value) than the medial electrode site, *p*<0.01 (see Figure 7). Furthermore, the attentional load by block-order interaction was not significant, *F*(3,75) = 1.22, *p* = 0.308, *η* ^2^ = 0.047, as was the electrode site by block-order interaction, *F*(2,50) = 0.747, *p* = 0.479, *η* ^2^ = 0.029, the attentional load by electrode site interaction, *F*(6,150) = 0.795, *p* = 0.523, *η* ^2^ = 0.031, and the interaction between attentional load, electrode site, and block-order, *F*(6,150) = 0.989, *p* = 0.413, *η* ^2^ = 0.038.

A RM-ANOVA was performed to look at the effect of attentional load, electrode site, and block-order on N100 latencies. There was no main effect of attentional load, *F*(3,75) = 0.588, *p* = 0.625, *η* ^2^ = 0.023, no main effect of electrode site, *F*(2,50) = 1.367, *p* = 0.265, *η* ^2^ = 0.052, and no main effect of block-order, *F*(1,25) = 2.225, *p* = 0.148, *η* ^2^ = 0.082. Furthermore, the attentional load by block-order interaction was not significant, *F*(3,75) = 1.061, *p* = 0.371, *η* ^2^ = 0.41, nor was the electrode site by block-order interaction, *F*(2,50) = 2.644, *p* = 0.081, *η* ^2^ = 0.096, the attentional load by electrode site interaction, *F*(6,150) = 1.534, *p* = 0.195, *η* ^2^ = 0.058, and the interaction between attentional load, electrode site, and block-order, *F*(6,150) = 1.649, *p* = 0.164, *η* ^2^ = 0.062.

#### P200

A RM-ANOVA was conducted to look at the effect of attentional load, electrode site, and block-order on P200 amplitudes. There was no main effect of attentional load, *F*(3,75) = 0.603, *p* = 0.615, *η* ^2^ = 0.024, no main effect of electrode site, *F*(2,50) = 2.013, *p* = 0.144, *η* ^2^ = 0.075, and no main effect of block-order, *F*(1,25) = 0.016, *p* = 0.9, *η* ^2^ = 0.001. The attentional load by block-order interaction was nonsignificant, *F*(3,75) = 0.055, *p* = 0.983, *η* ^2^ = 0.002, as was the electrode site by block-order interaction, *F*(2,50) = 2.259, *p* = 0.115, *η* ^2^ = 0.083, the attentional load by electrode site interaction, *F*(6,150) = 0.311, *p* = 0.93, *η* ^2^ = 0.012, and the three-way interaction between attentional load, electrode site, and block-order, *F*(6,150) = 1.354, *p* = 0.237, *η* ^2^ = 0.051.

Similar results were obtained from the RM-ANOVA performed to determine whether an effect of attentional load and electrode-site, with block-order as a between-subjects factor, existed for the latency of the P200. There was no main effect of attentional load, *F*(3,75) = 2.066, *p* = 0.112, *η* ^2^ = 0.076, no main effect of electrode site, *F*(2,50) = 0.991, *p* = 0.378, *η* ^2^ = 0.038, and no main effect of block-order *F*(1,25) = 1.181, *p* = 0.288, *η* ^2^ = 0.045. Furthermore, the attentional load by block-order interaction was not significant, *F*(3,75) = 1.294, *p* = 0.283, *η* ^2^ = 0.049, as was the electrode site by block-order interaction, *F*(2,50) = 0.204, *p* = 0.816, *η* ^2^ = 0.008, the attentional load by electrode site interaction, *F*(6,150) = 0.369, *p* = 0.898, *η* ^2^ = 0.015, and the interaction between attentional load, electrode site, and block-order, *F*(6,150) = 0.802, *p* = 0.57, *η* ^2^ = 0.031.

## Discussion

The aim of this study was to investigate whether increases in attentional load modulate vocal and neural responses to FAF perturbations. Participants produced vocalizations while exposed to FAF in both single and dual-task conditions. To manipulate participants’ attentional load, participants produced vocalizations while they either passively viewed a RSVP of letters, or while they attended to a RSVP of letters that was either presented at a low, intermediate, or high rate, in order to later identify target stimuli. A main effect of attentional load on both white letter identification accuracy, and “X”-placement accuracy was found, as target identification was better following the dual-task low and dual-task intermediate attentional load trials, relative to the dual-task high attentional load trials. These results suggest that the participants’ attentional load was successfully manipulated by increasing the stimulus presentation rate. Despite the differences in accuracy found across the different presentation rates, even in the highest attentional load condition participants accurately identified the white letter 87.5% of the time, while the “X”-placement was correctly identified 78.2% of the time. The high level of accuracy found even in the most attentionally demanding condition, suggests that while the attentional load manipulation was successful, participants were still able to maintain a high level of performance.

Examination of speakers’ compensatory responses to the brief FAF perturbations revealed that vocal response magnitudes were modulated by an attentional load by block order interaction. Participants who were exposed to the single-task condition, prior to the dual-task condition, produced larger vocal responses in the single-task condition, relative to the dual-task condition. However, participants who were exposed to the dual-task condition, prior to the single-task condition, produced similar vocal responses in both conditions. This interaction suggests that when participants completed the single-task prior to the dual-task, they were able to passively view the visual stream during the single-task, with sufficient attentional resources remaining to monitor their auditory feedback and correct for production errors. In contrast, when attentional resources were then split between the vocalization task and the target identification task, fewer attentional resources were available for participants to monitor their auditory feedback, resulting in smaller vocal responses. Although divided attention modulated responses to FAF when the single-task condition occurred prior to the dual-task condition, this was not the case when the dual-task condition occurred prior to the single-task condition. When the dual-task condition was completed prior to the single-task condition, vocal responses in the single-task condition were no different than those in the dual-task condition. We suggest that when participants were exposed to the dual-task, prior to the single-task, the single-task demanded more attentional resources as participants were unable to passively view the visual stream. Previous research has shown that extensive and consistent training on a task can lead to automatic processing of stimuli [37]. We suggest that after exposure to the dual-task, despite no longer being required to identify the target stimuli, participants continued to actively monitor the visual stream for targets, which resulted in fewer attentional resources for monitoring their auditory feedback. As a result, both the single-task and the dual-task resulted in divided attention, making vocal responses across these conditions indistinguishable. Although there was no effect of divided attention when the dual-task occurred first, the fact that vocal responses were larger in the single-task condition, when the single-task occurred first, suggests that divided attention modulates vocal responses to FAF.

The results of this study suggest that when attention is divided, smaller vocal responses to FAF are produced. One possible explanation for these smaller responses is that under divided attention auditory feedback is less salient. Previous research has shown that under visual attentional load, the loudness of tones is attenuated by 7 dB [38], which may be a consequence of reduced cochlear sensitivity [39], or decreased activation of the auditory cortex (e.g., Johnson & Zatorre, [40]). Furthermore, focused attention to an auditory channel has been associated with a 10 dB increase in loudness relative to an unattended auditory channel [25]. If divided attention decreased participants’ perception of the loudness of their auditory feedback, it is likely that the FAF perturbations became less salient, which resulted in smaller compensatory responses.

Although the size of the vocal responses to FAF were modulated by divided attention, vocal response latencies were not. We hypothesized that increasing attentional load would increase the processing demands of the visual task, and reduce the amount of attentional resources allocated to the processing of auditory feedback, resulting in later vocal responses. Although it is possible that vocal response latency is not affected by divided attention, it is also possible that the attentional load manipulation was not strong enough to affect the vocal response latencies recorded in this study. As previously mentioned, response accuracy was quite high, even for the highest level of attention load. This being said, while it appears as though attention can be divided without influencing the timing of vocal responses, this may not be the case in situations where attentional resources are more heavily taxed.

Divided attention resulted in the modulation of compensatory vocal responses to FAF; however, divided attention did not modulate the amplitude or latency of the P1-N1-P2 ERP components elicited by the FAF in this study. Although vocal responses were smaller under divided attention, they were still produced. This suggests that the FAF perturbations were detected by the auditory system in both the single- and dual-task conditions. Based on these results, we suggest that the effect of attentional load was not on the auditory processing of FAF, but instead it interfered with the integration of auditory and motor information, or motor control itself. It has previously been suggested that speech motor control may be disrupted under divided attention. For example, when participants were required to read sentences while also completing a secondary motor task, divided attention resulted in decreased displacement and velocity of labial movements [41], as well as increased sound pressure level [42]. Together with the results of this study, these findings suggest that speech motor control is susceptible to divided attention.

Even though divided attention was not found to modulate ERP responses to FAF, the amplitude of the N1 component was found to vary as a function of electrode site. N1 amplitudes were smaller (absolute value) at the right electrodes sites, relative to medial electrode sites. This result is unsurprising, as N1 responses elicited by FAF are generally largest at medial sites [18], [22].

Much like the size of the ERPs elicited by the FAF perturbations, and the vocal response latencies, the latencies of the ERP components were not found to differ when attention was divided. We hypothesized that divided attention may result in later ERP latencies as a result of increased processing demands. Although this hypothesis was not confirmed, it is possible that the dual-task condition was not demanding enough to increase processing demands to the extent that ERP latencies were affected. As mentioned previously, even during the most demanding attentional load condition, accuracy was still quite high, thus a more demanding task may be required to produce changes in ERP latencies as a function of attentional load.

Fluent speech production relies on auditory feedback for the regulation of ongoing and future speech motor commands. The aim of the current study was to investigate how attention modulates the processing of auditory feedback during ongoing speech. The results of this study suggest that divided attention can reduce the size of compensatory motor responses to FAF. However, the results of this study also suggest that the P1-N1-P2 ERP components elicited by FAF are less sensitive to divided attention. While the attentional load manipulation utilized in this study was successful at reducing target identification accuracy as the attentional load increased, accuracy was still quite high, even at the highest level of attentional load. With this being said, it is possible that auditory-cortical responses are less sensitive to increases in attention load, but may show attentional modulation if a secondary task is utilized that imposes a higher attentional load. Alternatively, sensorimotor integration, or motor control itself, may be more susceptible to increases in attentional load.

Throughout a typical day, we often encounter situations where we must speak while also performing other tasks. Recently, there has been a focus on assessing the impact of conversation on the performance of secondary tasks such as driving. We instead assessed the impact of divided attention on low level control of speech and found that our ability to use auditory feedback to monitor and regulate our speech may be compromised during many of our daily encounters. While typically developed adults have internal models that can execute fluent speech in a feedforward manner, previous research has suggested that children rely more on auditory feedback [19]. Future research should address how increases in attentional load may modulate sensory-motor integration, and speech motor control in this population, particularly in young children where auditory feedback may be particularly important for acquiring speech [2]. Furthermore, previous research has suggested that individuals who stutter may have an overreliance on auditory feedback [2]. Future research should assess stuttering severity under conditions of divided attention in these individuals, as divided attention may reduce their reliance on auditory feedback, and help to promote fluent speech.
